# An Unusual Diagnosis of Inguinal Pain in a Childbearing-Age Woman: An Inflamed Canal of Nuck Cyst With an Abnormal Leiomyomatous Proliferation

**DOI:** 10.7759/cureus.109199

**Published:** 2026-05-19

**Authors:** Ahmad El Khatib, Mohamad Yahfouf, Ali Hteit, Mohamad El Haress, Karim Kheir, Najwa Radwan, Najla Fakhruddin, Arwa Alzaghal, Mustapha El Lakis

**Affiliations:** 1 Faculty of Medicine, American University of Beirut, Beirut, LBN; 2 Faculty of Medicine and Medical Sciences, Georgian American University, Tbilisi, GEO; 3 Department of Surgery, American University of Beirut Medical Center, Beirut, LBN; 4 Department of Pathology and Laboratory Medicine, American University of Beirut Medical Center, Beirut, LBN; 5 Department of Radiology, American University of Beirut Medical Center, Beirut, LBN; 6 Department of Surgery, American University of Beirut, Beirut, LBN

**Keywords:** canal of nuck cyst, endometriosis, groin pain, inguinal canal, leiomyomatous proliferation

## Abstract

The canal of Nuck cyst is a rare pathology seen in women in the inguinal canal. It is often mistaken for a hernia or a lipoma; however, diagnosis is confirmed on surgical exploration and histopathological examination.

We present a case of a 38-year-old woman, gravida 2 para 2, with progressively worsening left lower quadrant abdominal and groin pain over two months. Imaging identified a small cystic structure in the canal of Nuck that had increased in size and demonstrated surrounding inflammatory changes. She underwent a laparoscopic transabdominal preperitoneal exploration; the canal lesion was excised, and the associated inguinal hernia was repaired. Histopathological findings were consistent with leiomyomatous proliferation.

We report a rare case of severe groin pain in a childbearing-age woman to highlight the differential diagnosis and describe this unusual finding. Documenting such cases is important for improving our understanding of canal of Nuck cysts and their pathogenesis.

## Introduction

A canal of Nuck cyst is an uncommon lesion of the female inguinal canal that extends in the direction of the labia majora [1]. It is an embryological remnant that represents the counterpart of the male processus vaginalis and, analogous to that structure, may present with a hydrocele or cyst [2]. Clinically, patients often present with an inguinal swelling, lipoma, lymphadenopathy or an abscess until a definitive diagnosis is made by histopathology [3]. There are several types of canal of Nuck pathology including an encysted mass, a communicating hydrocele, and an hourglass type. While more commonly found to be lined with mesothelial cells as it is considered a peritoneal extension, cases with endometriosis have been described, and exceedingly rare reports document leiomyomatous proliferation within the cyst [4,5]. We present the case of a canal of Nuck cyst containing leiomyomatous proliferation to underscore the importance of thorough pathological assessment of excised lesions, particularly in patients with relevant comorbidities such as endometriosis and polycystic ovary syndrome. To date, around 400 case reports of canal of Nuck pathology exist in the literature, with cases of leiomyomatous proliferation virtually not reported, which highlights the novelty and importance of this case [6]. There is no consensus on the prevalence of canal of Nuck cysts, with estimates from multiple studies ranging from 0.74% to 1% in young women who had an inguinal swelling, with no data on adults [2].

## Case presentation

A 38-year-old woman, gravida 2 para 2 via cesarean sections, with a history of polycystic ovary syndrome and endometriosis, presented to the emergency department with severe pain in her left lower abdomen and left groin. The patient stated that the pain began one week prior to presentation and progressively increased in intensity up to the day of presentation. On that day, the pain gradually worsened and migrated from both lower pelvic areas to the left groin. It became constant, very severe (10/10), and did not improve with pain medication. She denied nausea, vomiting, recent change in bowel habits, fever, chills, or urinary symptoms. She also reported regular and normal menstrual cycles, with her last menstrual cycle being 2-3 weeks ago.

Physical exam was consistent with marked tenderness in the left groin and lower abdomen, and a cystic swelling in the left inguinal canal in the standing position. There was no overlying skin redness, warmth, or discharge. Her WBC count was at 7500/mm^3^, with 74% neutrophils and 21% lymphocytes.

**Figure 1:**
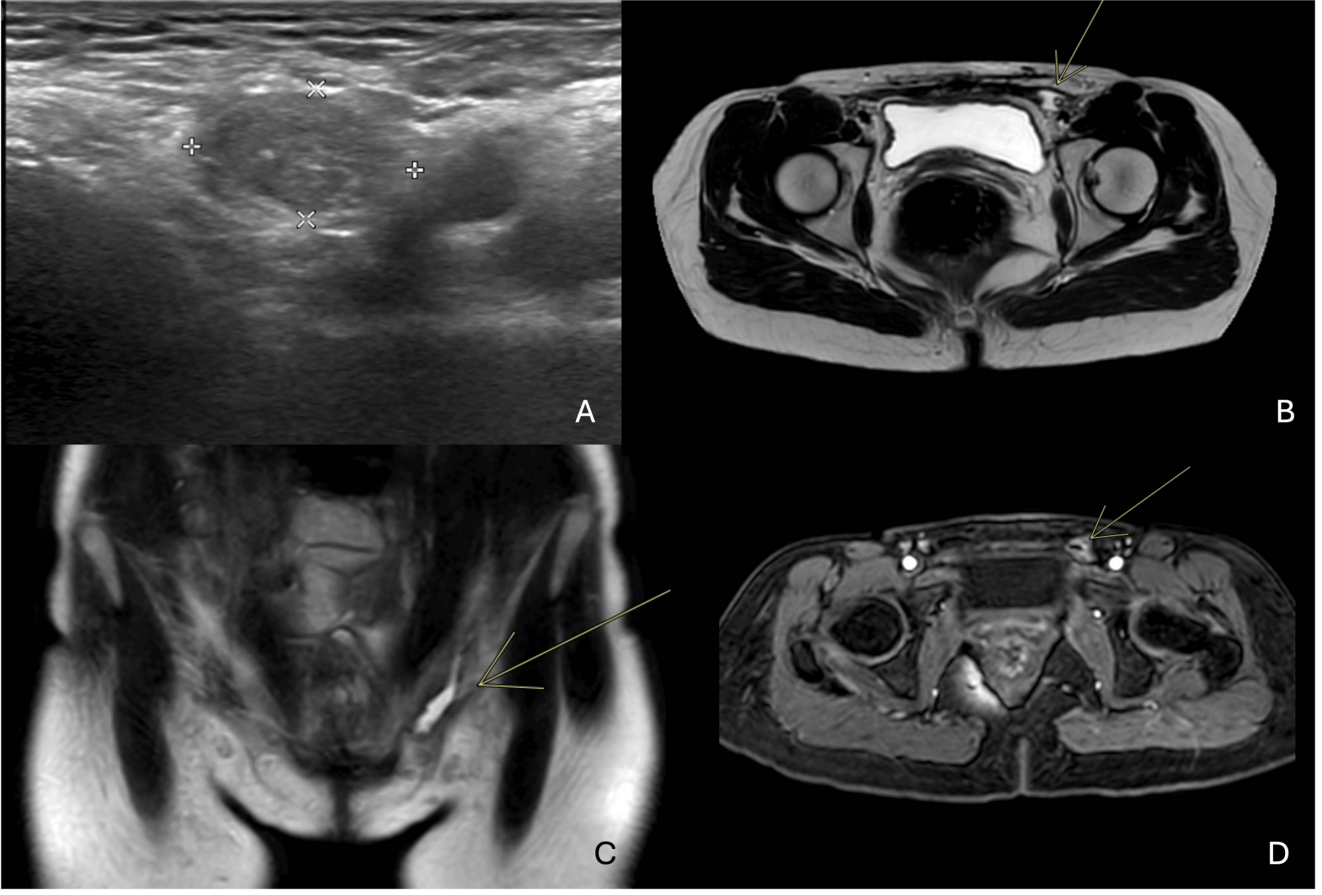
(A) A 1.8 × 1.1 cm oval, well-defined mildly hyperechoic structure is identified in the left inguinal region, located medial to the femoral artery with no internal vascularity. Axial (B) and coronal (C) T2-weighted images demonstrate a well-defined high T2 signal/cystic lesion in the left inguinal region extending toward the proximal portion of the canal of Nuck. (D) In the axial post-contrast T1-weighted image, there is mild peripheral wall thickening and enhancement suggesting a small canal of Nuck cyst with mild associated inflammation.

An earlier ultrasound in October 2025 demonstrated an echogenic, heterogeneous, well-defined vascularized mass medial to the left common femoral artery, measuring 1.8 × 1.1 cm, which prompted further evaluation (Figure 1A). A subsequent magnetic resonance imaging (MRI) identified a small cystic structure in the left inguinal region at the proximal canal of Nuck, measuring approximately 0.9 × 0.8 cm. The lesion had high signal intensity on T2-weighted imaging and low signal intensity on T1, with mild peripheral wall thickening and enhancement, but no diffusion restriction (Figures 1B, 1C, 1D). No suspicious soft tissue mass was seen, and pelvic organs appeared normal. These findings suggested a canal of Nuck cyst with inflammatory features, and interval follow-up was recommended.

**Figure 2:**
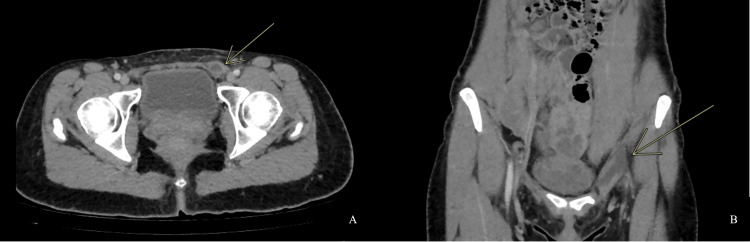
Axial (A) and coronal (B) images show a mild interval increase in the size of the left canal of Nuck cyst with increased peripheral wall enhancement and surrounding inflammatory fat stranding, suggestive of a superimposed infectious process.

At the current presentation, a contrast-enhanced CT scan showed interval enlargement of the cystic structure in the left inguinal canal to 1.7 × 1.2 cm, with increased peripheral wall enhancement, surrounding fluid, and inflammatory changes (Figure 2). These findings raised concern for a superimposed infectious or inflammatory process.

**Figure 3:**
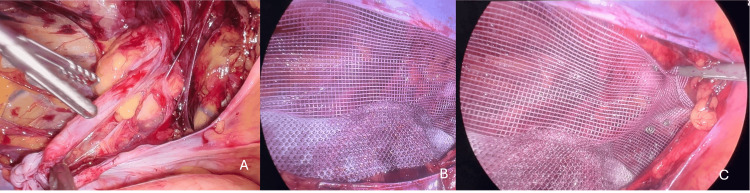
(A) Left indirect inguinal cyst arising from the round ligament was identified occupying the inguinal canal. (B) Parietex™ TECT1510AL polyester mesh (15 × 10 cm). (C) Protack fixation device 5mm AUTOSUTURE 174006.

The patient underwent a laparoscopic transabdominal preperitoneal exploration of the left groin with cyst excision and repair of the inguinal hernia with Parietex™ TECT1510AL polyester mesh (15 × 10 cm). Intraoperatively, a left indirect inguinal cyst arising from the round ligament was identified occupying the inguinal canal (Figure 3A). The round ligament and cyst were excised en bloc with proximal ligation. Also identified was a direct inguinal hernia that contained a dark chocolate-colored cystic lesion, which was excised. Mesh repair with Parietex™ TECT1510AL polyester mesh (15 × 10 cm) (Figure 3B) was done and fixed with Protack fixation device 5mm AUTOSUTURE 174006 (Figure 3C). Specimens were retrieved using an endoscopic catch bag, and the procedure was completed without any complications.

**Figure 4:**
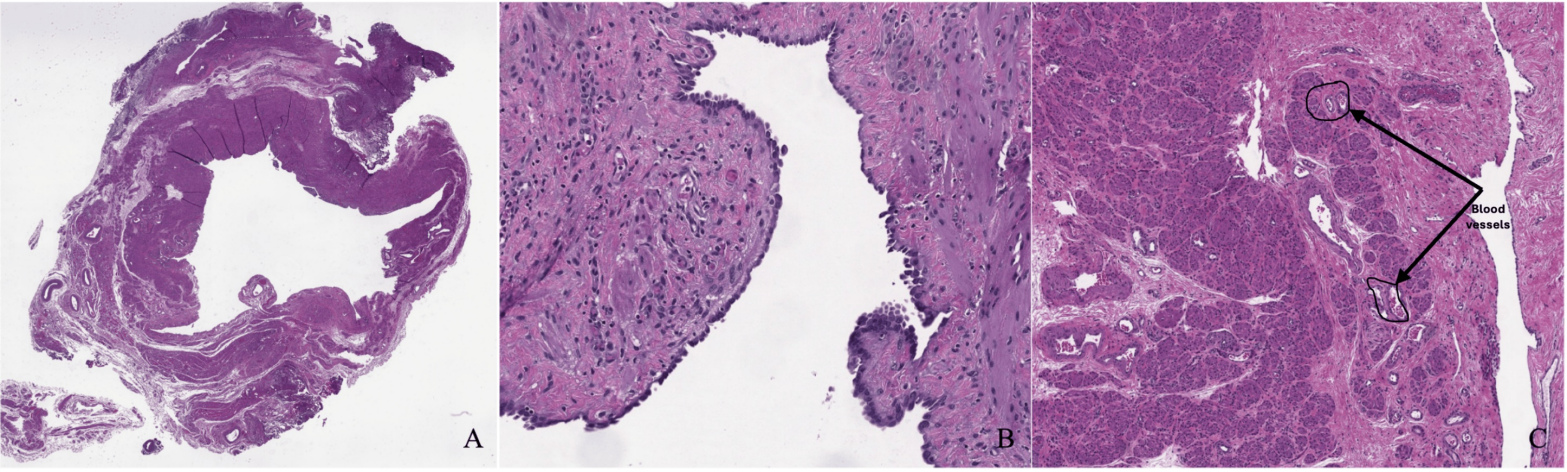
Histologic examination reveals a cystic lesion (a), lined by a single layer of flat to cuboidal mesothelial cells (b). The cyst is surrounded by a proliferation of smooth muscles and blood vessels (c). However, no features suggestive of malignancy are identified.

The post-operative course was unremarkable. At the two-week follow-up, the patient was clinically stable and reported no residual symptoms. Histopathological examination showed fragments of fibro-membranous tissue, some covered by adipose tissue. The microscopic examination revealed a hernia sac with two benign lymph nodes inside. In addition, a mesothelial-lined cyst surrounded by smooth muscle proliferation and vascular elements was found. The lesion had benign histological characteristics and no evidence of malignancy was identified. There was evidence of atypically proliferated smooth muscle bundles around vascular walls as well as extending into adjacent skeletal muscle (Figure 4). These findings supported a diagnosis of a benign canal of Nuck cyst with smooth muscle proliferation; differential diagnoses could have included angioleiomyoma and a small perivascular epithelioid cell tumor.

## Discussion

The canal of Nuck is a persistent peritoneal extension accompanying the round ligament through the inguinal canal towards the labia majora. Normally, this embryologic structure undergoes obliteration during infancy; however, failure of this process may result in persistence of the canal, which may predispose to indirect inguinal hernia or fluid accumulation leading to a hydrocele or a cyst of the canal of Nuck. Although inguinal hernias are common surgical conditions, cystic lesions of the canal of Nuck remain rare [3,7].

Patients with canal of Nuck cysts typically present with inguinal swelling or groin pain, and the lesion may clinically mimic other conditions such as inguinal hernia, lymphadenopathy, lipoma, abscess, or soft-tissue tumors (Table 1) [8]. Because of this nonspecific clinical presentation, imaging plays an important role in evaluating these lesions. Ultrasonography is usually the first diagnostic modality, while MRI provides better characterization of the cystic lesion and its relationship to the inguinal canal and surrounding structures.

**Table 1:** Differential diagnosis for inguinal pain in women of childbearing age.

Type of Pathology	Differential Diagnosis
Hernia	Inguinal hernia
	Femoral hernia
Congenital	Canal of Nuck cyst
Lymphatic	Lymphadenopathy of the inguinal area
Infectious	Abscess
Gynecologic	Inguinal endometriosis
	Round ligament varices
	Referred pain from the ovaries, Bartholin cyst
Vascular	Saphena varix
	Femoral artery aneurysm
Neoplasm	Sarcoma
	Lipoma
Other	Psoas abscess
	Musculoskeletal causes

In our patient, ultrasonography demonstrated a heterogeneous vascularized lesion medial to the left common femoral artery, prompting further evaluation with an MRI, which demonstrated a small cystic structure located along the proximal portion of the left canal of Nuck measuring approximately 0.9 × 0.8 cm. Mild peripheral wall thickening and enhancement suggested an inflammatory component, which are findings consistent with the previously reported imaging characteristics of canal of Nuck cysts [9].

Previously reported cases of canal of Nuck cysts are limited mainly to isolated case reports and small case series. Fewer than several hundred cases of canal of Nuck hydroceles and cysts have been reported worldwide, highlighting the potential gap in our knowledge of these lesions. A case series by Adhikari and Bhatta [10] showed the young age of discovery of these lesions, ranging from 1 to 6 years of age, with most of them reporting painless swelling and management being surgical excision. In our case, the patient reported severe pain in her groin, which highlights the uniqueness of her presentation.

Furthermore, these lesions are more commonly discovered in childhood but could persist into adulthood with no manifestations. Most reported patients present with inguinal swelling and are initially suspected to have inguinal hernias, with the final diagnosis often being established only after surgical exploration. These lesions are typically located within the inguinal canal, occasionally extending toward the labia majora, and may be associated with the round ligament or inguinal hernias. Surgical excision remains the treatment of choice in most reported cases, with both open and laparoscopic approaches described depending on the clinical presentation and associated pathology (Table 2) [11,12].

**Table 2:** Comparison between open and laparoscopic surgical approaches for canal of Nuck cysts

Features	Open Approach	Laparoscopic Approach
Approach	External groin incision	Intra-abdominal or preperitoneal
Blood loss	High	Lower risk
Post-op pain	More pain	Less pain
Completeness of excision	May miss proximal extension, and cysts may rupture if incarcerated	Better for identification of the entire cyst
Visualization	Limited/Local	Superior visualization and can assess both sides
Recovery duration	Longer hospital stay	Faster recovery
Cosmetic appearance	Visible scar	Better outcomes
Wound complications	High risk	Lower risk
Concurrent hernia repair	Limited	Can perform hernioplasty

Laparoscopic exploration identified a left indirect inguinal cyst arising from the round ligament and occupying the inguinal canal. In addition, a direct inguinal hernia was identified, within which a dark chocolate-colored cystic lesion was found and excised. The cyst showed no signs of infection and was excised intact without rupture or spillage of contents.

Histopathologic examination demonstrated a mesothelial-lined cyst with smooth muscle proliferation and benign histologic morphology. Expert pathological consultation further described atypical leiomyomatous-type smooth muscle proliferation surrounding vascular structures. While these findings support a cyst of the canal of Nuck, the morphologic features also raised a differential diagnosis including angioleiomyoma and perivascular epithelioid cell tumor (PEComa). The lesion demonstrated no cytologic atypia, mitotic activity, or other features suggestive of malignancy. The presence of a mesothelial-lined cyst associated with smooth muscle proliferation is an unusual finding and expands the histopathologic spectrum of lesions involving the canal of Nuck.

Another noteworthy aspect of this case is the patient’s known history of endometriosis and the intraoperative identification of a dark chocolate-colored cystic lesion. Such a gross appearance raises the possibility of hemorrhagic change and highlights the importance of considering extra-pelvic endometriosis in the differential diagnosis of inguinal masses. Endometriosis involving the inguinal canal or round ligament can present with painful groin swelling mimicking an inguinal hernia [5,13]. Although histologic evidence of endometriosis was not identified in this specimen, the clinical context and operative findings emphasize the importance of considering this entity.

Overall, this case illustrates the diagnostic challenges associated with inguinal masses in female patients. Canal of Nuck cysts remain a rare entity and may coexist with other inguinal pathologies including hernias or unusual mesenchymal lesions. Surgical excision therefore serves both as a diagnostic and a therapeutic purpose, allowing definitive treatment and accurate diagnosis.

## Conclusions

We report a rare case of a mesothelial-lined cyst with smooth muscle proliferation associated with a cyst of the canal of Nuck and concomitant inguinal hernia. The combination of unusual histopathologic findings, involvement of the round ligament, and the patient’s clinical background makes this presentation particularly noteworthy.
